# Disparities in Receipt of Advice to Quit Smoking From Health Care Providers: 2010 National Health Interview Survey

**DOI:** 10.5888/pcd11.140053

**Published:** 2014-07-31

**Authors:** David Danesh, Electra D. Paskett, Amy K. Ferketich

**Affiliations:** Author Affiliations: David Danesh, Electra D. Paskett, Amy K. Ferketich, The Ohio State University, Columbus, Ohio.

## Abstract

**Introduction:**

Not all smokers receive tobacco cessation advice from health care providers (HCPs) and, although factors associated with receiving HCP advice to quit smoking and the effectiveness of such advice have been examined, no recent study has explored differences between types of HCPs (eg, physicians vs dentists). Our objective was to determine the prevalence of HCP-delivered advice and the characteristics of patients who receive advice to quit smoking from any HCP and, separately, from a physician or a dentist.

**Methods:**

This study used data from the Sample Adult Core questionnaire, Sample Family Core questionnaire, and Sample Adult Cancer Control Module of the 2010 National Health Interview Survey. The sample for the analysis was limited to current smokers who saw an HCP in the previous 12 months. The characteristics of smokers who received advice to quit were compared with those who did not receive advice and further analyzed by which type of HCP delivered the advice.

**Results:**

Half of current smokers reported receiving advice to quit smoking from any HCP, but only 1 in 10 smokers who visited a dentist received advice to quit. Receipt of advice was associated with sex, age, race, marital status, region, type of health insurance, quit attempts in the previous 12 months, and extent of tobacco use.

**Conclusion:**

Only half of all smokers receive advice to quit from any HCP and even fewer from dentists. Changes in professional organizations’ policies, health profession education curriculum, and continuing education requirements are needed to improve compliance with the Clinical Practice Guideline.

## Introduction

Cigarette smoking and secondhand smoke exposure result in approximately 443,000 premature deaths annually, making it the leading cause of preventable death in the United States (1). In 2011, it was estimated that 43.8 million, or 19.0%, of US adults were current cigarette smokers (2). Tobacco use harms nearly every organ of an individual’s body, leading to multiple chronic respiratory and cardiovascular health complications, as well as oral diseases such as leukoplakia, periodontitis, and a variety of cancers, including oral cancer (3).

The US Public Health Service (PHS) developed the Tobacco Use and Dependence Clinical Practice Guideline, recommending that all health care providers (HCPs) promote tobacco cessation to all patients (4). All clinicians, including dental professionals, should acknowledge the health complications of smoking and follow PHS guidelines to promote tobacco use cessation to all patients to improve patient health. However, the evidence shows that not all HCPs advise patients to quit smoking (5–10). Moreover, HCPs may overestimate the amount of advice they give to patients (5). Data from the 1992 National Health Interview Survey suggested that 52% of smokers reported they were advised by a physician to quit, whereas only 24% reported they were advised to quit by a dentist (6). A national survey of HCPs during 2003–2004 found that 94.9% of primary care physicians, 70.6% of dentists, and 77.5% of dental hygienists reported they regularly advised their patients to stop smoking (5). Although the reporting periods differed by 10 years, most of the difference in rates of advice is likely the result of differences in reporting by patients and HCPs.

HCPs cite barriers to delivering tobacco cessation advice, including lack of knowledge about tobacco cessation treatment effectiveness and delivery, time constraints, lack of cessation intervention training, and lack of patient insurance reimbursement or payment for treatment (4). Previous reports show that patient sociodemographic and tobacco status characteristics are associated with whether a patient receives tobacco use cessation advice from HCPs (6,8–10). To our knowledge, little research has examined the extent to which dentists provide advice to quit smoking, and whether certain patients are more or less likely to receive such advice. The objective of this study was to determine the prevalence of HCP-delivered advice to quit smoking and the characteristics of patients associated with the receipt of cessation advice from physicians and, separately, from dentists.

## Methods

This study involved a cross-sectional secondary analysis of data from the 2010 National Health Interview Survey (NHIS). The NHIS annually collects data related to the health of the civilian, noninstitutionalized, household population of the United States (11). Data were collected through personal household interviews conducted by interviewers trained by the US Bureau of the Census. The survey sampling plan provides for a representative sampling of the population by using a multistage area probability design. Overall, blacks, Hispanics, and Asians were oversampled by screening and sampling areas with higher concentrations of these groups. The 2010 NHIS sample included 34,329 households, yielding 89,976 persons in 35,177 families, which represented an overall household response rate of 79.5%. One randomly selected adult aged 18 or older from each family interviewed was asked the Sample Adult Component and Cancer Control Module questions, which yielded a total sample size of 27,157 persons and a 60.8% response rate.

The sample for the analysis was limited to participants who self-reported they were current smokers (defined as those who smoke every day or some days and have smoked 100 or more cigarettes in their lives) and who had seen any HCP within the previous 12 months. Because data from the Sample Adult Core questionnaire, Sample Family Core questionnaire, and Sample Adult Cancer Control Module were used, only adults aged 18 or older were included in the analysis, and merged from each data set.

### Measures

The 3 primary dependent variables were receipt of advice to quit (yes or no) by any HCP, by a doctor, or by a dental worker (dentist or dental hygienist). These variables were measured by asking participants, “In the past 12 months, has a medical doctor, dentist, or other health professional advised you to quit smoking or quit using other kinds of tobacco?” Then, participants would specify whether a doctor, dentist, dental hygienist, or other health professional advised them to quit smoking or using other kinds of tobacco. We merged dentists and dental hygienists into one category: dental worker.

The primary independent variables in this study were the sociodemographic variables of sex, age, race/ethnicity, marital status, region, education, health insurance type, poverty status and the tobacco-related variables of daily tobacco use and quit attempts in the previous 12 months.

We estimated the prevalence of receiving advice to quit from any HCP and, separately, from a doctor or a dental worker. Next, the prevalence of receiving advice from any HCP, a doctor, or a dental worker was examined by sociodemographic characteristics and HCP type. Stata 13 (StataCorp) was used for the analysis. Prevalence estimates, 95% confidence intervals, and *P* values for tests of differences in proportions were estimated by using the weights and other survey design features of the NHIS.

## Results

We analyzed data on 5,147 current smokers, of whom 3,612 had seen an HCP within 12 months of the survey. Approximately half of the participants in the sample were female; most were aged 25 to 64 years (77.8%) and non-Hispanic white (76.2%) (Table 1).

**Table 1:** Adult US Smokers Who Visited a Health Care Provider and Received Advice to Quit, 2010 National Health Interview Survey

Characteristics	Weighted % in Sample (n = 3,612^a^)	Prevalence of Receiving Advice (95% CI)	*P* Value
Overall	100	54.4 (52.4–56.4)	NS^b^
**Sex**			
Male	47.4	52.1 (49.2–54.9)	.0230
Female	52.6	56.5 (53.8–59.2)	
**Age, y**			
18–24	12.2	38.6 (32.9–44.6)	<.0001
25–44	38.3	50.8 (47.6–54.1)	
45–64	39.5	61.2 (58.3–64.0	
≥65	10.1	60.7 (55.6–65.6)	
**Race/ethnicity **			
Non-Hispanic white	76.2	56.6 (54.2–59.0)	<.0001
Non-Hispanic black	12.5	49.7 (45.1–54.4)	
Hispanic or Latino	8.1	41.0 (35.5–46.7)	
Other races, non-Hispanic	3.3	55.3 (44.1–65.9)	
**Marital status**			
Never married	22.1	46.5 (42.4 –50.7)	<.0001
Married/living with partner	56.9	56.1 (53.4–58.7)	
Divorced/widowed/separated	21.1	58.4 (55.2–61.4)	
**Geographic region^c^**			
Northeast	16.3	61.1 (55.7–66.2)	.0013
Midwest	27	57.5 (53.7–61.2)	
South	38.9	50.6 (47.3–53.8)	
West	17.8	52.0 (47.5–56.5)	
**Education level**			
Less than high school	18.1	56.2 (51.8–62.6)	NS
High school graduate	28	52.5 (48.4–56.6)	
GED	6.7	56.8 (49.2–64.0)	
Some college	33.5	55.1 (52.0–58.2)	
College grad or higher	13.7	53.8 (48.6–58.9)	
**Health insurance type **			
Any Medicaid	14.2	58.0 (53.3–62.6)	<.0001
Medicare, no Medicaid	12	64.0 (59.1–68.7)	
Any other insurance	52.5	56.6 (53.7–59.5)	
Uninsured	21.3	41.5 (37.1–46.0)	
**Poverty status**			
Below poverty line	17.8	50.8 (46.4–55.1)	NS
Above poverty line	75.1	55.7 (53.3–58.1)	
Missing data	7.2	50.1 (42.0–58.2)	
**Any quit attempts in previous 12 months **			
Yes	47.9	57.4 (54.7–60.1)	.0023
No	52.1	51.7 (48.8–54.5)	
**Tobacco consumption**			
Some days	21	36.6 (32.5–40.9)	<.0001
1-10 CPD	36.3	54.6 (51.4–57.8)	
11 or higher CPD	42.7	63.1 (60.1–66.1)	

Abbreviations: CI, confidence interval; NS, not significant; GED, general educational development; CPD, cigarettes per day.

a Not all participants answered all questions; some percentages may not total 100.

b Some *P* values are missing because they were not statistically significant.

c **Northeast:** Connecticut, Maine, Massachusetts, New Hampshire, New Jersey, New York, Pennsylvania, Rhode Island, and Vermont; **Midwest:** Illinois, Indiana, Iowa, Kansas, Michigan, Minnesota, Missouri, Nebraska, North Dakota, Ohio, South Dakota, and Wisconsin; **South:** Alabama, Arkansas, Delaware, District of Columbia, Florida, Georgia, Kentucky, Louisiana, Maryland, Mississippi, North Carolina, Oklahoma, South Carolina, Tennessee, Texas, Virginia, and West Virginia; **West:** Alaska, Arizona, California, Colorado, Hawaii, Idaho, Montana, Nevada, New Mexico, Oregon, Utah, Washington, and Wyoming.

Overall, 54.4% of current smokers who had seen an HCP within 12 months of the survey stated that they had received advice to quit smoking or using other kinds of tobacco in the past 12 months (Table 1). Participants who were male, aged 18 to 24, Hispanic, had never married, were from the Southern region of the United States, were uninsured, had no quit attempts in the preceding 12 months, and smoked some days were less likely than others in the same characteristic category to receive advice to quit by any HCP. Educational level and income status were not associated with receiving advice (Table 1).

Of current smokers who visited a doctor, 50.7% were advised to quit. Of current smokers who visited a dental worker, 11.8% were advised to quit (Table 2). Of all smokers who reported receiving advice to quit smoking, more than 90% reported receiving the advice from a physician, whereas only 13.5% reported receiving such advice from a dental worker.

**Table 2:** Adult US Smokers Who Visited a Health Care Provider and Received Advice to Quit, by Type of Health Care Provider, 2010 National Health Interview Survey

Characteristic	% Advice from Doctor (95% CI) (n = 3,611^a^)	*P* Value	% Advice from Dentist/Dental Hygienist (95% CI) (n = 2,187^b^)	*P* Value
**Sex**				
Male	48.4 (45.6–51.3)	.0270	12.8 (10.7–15.2)	NS
Female	52.7 (50.2–55.3)		10.9 (9.1–13.4)	
**Age, y **				
18–24	31.7 (26.2–37.9)	<.0001	9.5 (6.0–14.6)	NS
25–44	46.5 (43.5–49.6)		11.9 (9.6–14.6)	
45–64	58.3 (55.3–61.2)		13.6 (11.4–16.2)	
≥65	59.8 (54.7–64.6)		6.3 (3.4–11.3)	
**Race/ethnicity **				
Non-Hispanic white	52.6 (50.3–55.0)	.0002	13.0 (11.3–14.9)	.0113
Non-Hispanic black	46.9 (42.3–51.5)		9.0 (6.1–13.2)	
Hispanic or Latino	38.7 (33.4–44.3)		7.5 (4.4–12.5)	
Other races, non-Hispanic	50.0 (39.3–60.6)		6.3 (3.3–11.4)	
**Marital status **				
Never married	42.4 (38.4–46.6)	.0001	10.1 (7.6–13.3)	NS
Married/living with partner	52.2 (49.6–54.8)		12.7 (10.7–14.9)	
Divorced/widowed/separated	55.5 (52.3–58.7)		11.4 (9.0–14.5)	
**Geographic region^c^**				
Northeast	59.2 (53.9–64.2)	.0004	10.9 (7.8–14.9)	NS
Midwest	52.5 (49.1–55.9)		13.6 (11.3–16.2)	
South	47.3 (44.1–50.5)		10.4 (8.0–13.6)	
West	47.6 (43.0–52.3)		12.7 (9.7–16.3)	
**Education level**				
Less than high school	53.9 (49.5–58.3)	NS	9.7 (6.5–14.0)	NS
High school graduate	49.0 (44.9–53.1)		11.1 (8.7–14.0)	
GED	51.6 (44.3–58.9)		12.4 (6.8–21.5)	
Some college	51.0 (47.8–54.2)		13.3 (10.9–16.2)	
College grad or higher	49.5 (44.3–54.6)		11.5 (8.0–16.1)	
**Health insurance type **				
Any Medicaid	55.1 (50.6–59.5)	.0001	11.1 (7.0–17.2)	.0156
Medicare, no Medicaid	62.3 (57.4–66.9)		7.7 (4.6–12.8)	
Any other insurance	51.6 (48.7–54.4)		13.6 (11.7–15.8)	
Uninsured	39.5 (35.2–44.0)		7.9 (5.5–11.1)	
**Poverty status**				
Below poverty line	47.2 (42.8–51.6)	NS	10.7 (7.3–15.5)	NS
Above poverty line	51.9 (49.6–54.2)		12.5 (10.8–14.3)	
Missing data	47.2 (39.4–55.1)		7.0 (3.7–12.9)	
**Any quit attempts in past 12 months **				
Yes	53.2 (50.5–55.8)	.0270	13.2 (11.1–15.6)	NS
No	48.5 (45.7–1.3)		10.7 (8.9–12.9)	
**Tobacco consumption **				
Some days	32.9 (29.0–37.1)	<.0001	6.2 (4.3–9.0)	<.0001
1–10 CPD	51.0 (47.8–54.1)		9.8 (7.8–12.3)	
≥11 CPD	59.4 (56.3–62.4)		17.6 (14.9–20.7)	

Abbreviations: CI, confidence interval; NS, not significant; GED, general education development diploma; CPD, cigarettes per day.

a Among current smokers who saw a doctor. Not all participants answered all questions; some percentages may not total 100.

b Among current smokers who saw a dentist or hygienist. Not all participants answered all questions; some percentages may not total 100.

c **Northeast:** Connecticut, Maine, Massachusetts, New Hampshire, New Jersey, New York, Pennsylvania, Rhode Island, and Vermont; **Midwest:** Illinois, Indiana, Iowa, Kansas, Michigan, Minnesota, Missouri, Nebraska, North Dakota, Ohio, South Dakota, and Wisconsin; **South:** Alabama, Arkansas, Delaware, District of Columbia, Florida, Georgia, Kentucky, Louisiana, Maryland, Mississippi, North Carolina, Oklahoma, South Carolina, Tennessee, Texas, Virginia, and West Virginia; **West:** Alaska, Arizona, California, Colorado, Hawaii, Idaho, Montana, Nevada, New Mexico, Oregon, Utah, Washington, and Wyoming.

We analyzed the prevalence of advice from doctors and dental workers by characteristics of the smoker (Table 2). Among current smokers who visited a doctor in the 12 months preceding the survey, those who were female, aged 65 or older, non-Hispanic white, divorced/separated/widowed, from the Northeast region of the United States, covered by Medicare only, tried to quit in the previous 12 months, or smoked more than a half a pack a day were more likely than others in the same characteristic category to receive advice to quit smoking or using tobacco.

The patient characteristics associated with receiving advice from dental workers were somewhat different. Dental workers were more likely to advise smokers who were male, aged 45 to 64, non-Hispanic white, and covered by insurance other than Medicare or Medicaid than to advise other patients in the same characteristic category. However, only the findings for race/ethnicity and health insurance type were significant. Consistent with overall HCP advising, dental workers were more likely to advise people who were the heaviest smokers to quit (Table 2).

## Discussion

Physicians and dental professionals are not advising all of their patients to quit smoking, as recommended by the Clinical Practice Guideline (4). Half of current smokers received advice to quit smoking from any HCP or a physician, while only 1 in 10 smokers who visited a dentist in the year before the 2010 NHIS reported receiving advice to quit from a dental worker. This finding suggests that dental professionals, in particular, are missing an opportunity to teach patients the benefits of quitting smoking and the consequences of continued smoking, particularly from an oral health perspective. Previous studies show that smokers are receptive to and even expect to receive tobacco cessation advice from dental workers in both private practice and academic dental clinic settings (12,13). All HCPs, including dental professionals, should advise smokers to quit smoking because any amount of advice is effective in improving long-term quit rates and because it is the responsibility of the HCP to improve the health of the patient (4). Why these differences exist is unclear. Among all age groups, smokers aged 65 or older received the most advice to quit from doctors, while this age group received the least advice from dentists. This difference could be explained by the 2005 expansion of Medicare to reimburse providers for tobacco cessation counseling and the fact that Medicare patients could have comorbid conditions caused by smoking (14). Both of these factors would cause individuals older than 65 to visit doctors at a higher rate and receive advice to quit. Educational level and income level did not play a significant role in advice delivery. One general explanation for these differences could be that physicians and dental professionals face different barriers to delivering cessation advice, ranging from lack of time to lack of proper training or education (15,16).

Our results can be compared with those of previous studies that examined HCP-delivered advice to quit tobacco use. Tomar and colleagues found that dentists give more advice to quit than we found (24.1% vs 11.8%) (6). In comparison, the prevalence of advice to quit by physicians was comparable to our result (51.6% vs 50.7%). Other studies on the topic reported a similar prevalence (17) or a slightly higher prevalence (7) of physician advising. What smokers report and what HCPs report with respect to delivering advice to quit varies widely. When HCPs rate themselves, the prevalence of delivering advice to quit tobacco use is much higher (65.6%–94.9%) (5). Whether or not smokers underreport receiving advice or HCPs overreport delivering advice, or a combination of both, is unknown.

The unique contribution of this study is the analysis of the factors associated with the delivery of advice by dental professionals. Although previous studies focused on physicians and any other provider as a general category (6,8–10), little was known about which types of smokers dental professionals were more likely to advise to quit. Our results suggest that smokers who have a socioeconomic advantage (non-Hispanic whites and individuals with private health insurance) are more likely than others to receive advice to quit tobacco by dental professionals. The results also suggest that heavy smokers are more likely than light smokers to receive advice.

This study’s strengths are that it uses recent data to provide a large-scale, population-based analysis of HCP-delivered advice to quit smoking in the United States. Additionally, this study builds on previous analyses of NHIS data to include different types of HCPs (5,6,8–10). A limitation of the study is that NHIS data are self-reported, and individuals may not remember or may refuse to report receipt of advice to quit by an HCP. Another limitation is that the outcomes of the receipt of advice, such as provider interventions or quit attempts, were not examined. The study is also cross-sectional, which does not allow for the analysis of trends in advising. We also could not consider the type of practice patients visited (eg, private practice, academic clinic). Finally, the possibilities for comparisons with previous studies may be limited because of changes in NHIS survey design and sampling procedures.

Our analysis showed that individuals who were male, aged 18–24 years, Hispanic or Latino, never married, from the Southern region of the United States, uninsured, had no quit attempts in the preceding 12 months, and smoked only some days were less likely to receive advice to quit by any HCP. Education level and poverty status were not associated with receiving advice from any HCP. Our findings also showed a disparity in delivery of tobacco cessation advice between doctors and dental workers, showing the need to emphasize the importance of dental workers advising all patients who smoke to quit. Doctor and dental worker delivery of tobacco cessation advice varied by patient characteristics: heavy smokers consistently received more advice from both types of HCPs. However, regardless of provider type, we also found that HCPs are either not advising all patients to quit smoking tobacco (as recommended in the Clinical Practice Guideline) (4) or their advice in not effective enough for patients to remember. Because any tobacco cessation advice is effective at increasing quit attempts by smokers, changes in the policies of professional organizations, the health profession education curriculum, and the continuing education requirements would be beneficial in promoting advice delivery by HCPs. Future research should evaluate tobacco cessation education for providers and pilot new provider education programs. Also, worth exploring are the barriers that prevent different types of HCPs from advising patients to quit tobacco use. Finally, examining the receptiveness of smokers to receiving tobacco cessation advice from different types of HCPs could be another topic for further research.
